# Adherence to the Mediterranean diet and depression, anxiety, and stress symptoms in Chilean university students: a cross-sectional study

**DOI:** 10.1590/0102-311XEN206722

**Published:** 2023-11-13

**Authors:** Gladys Morales, Teresa Balboa-Castillo, Rubén Fernández-Rodríguez, Miriam Garrido-Miguel, Camilo Molino Guidoni, Rafaela Sirtoli, Arthur Eumann Mesas, Renne Rodrigues

**Affiliations:** 1 Facultad de Medicina, Universidad de La Frontera, Temuco, Chile.; 2 Centro de Estudios Sociosanitarios, Universidad de Castilla-La Mancha, Cuenca, España.; 3 Facultad de Enfermería, Universidad de Castilla-La Mancha, Albacete, España.; 4 Universidade Estadual de Londrina, Londrina, Brasil.

**Keywords:** Mediterranean Diet, Depression, Mental Health, Young Adults, Student Health, Dieta Mediterránea, Depresión, Salud Mental, Adulto Joven, Salud del Estudiante, Dieta Mediterrânea, Depressão, Saúde Mental, Adulto Jovem, Saúde do Estudante

## Abstract

This study aims to determine the association of adherence to the Mediterranean diet and its food groups with depressive symptoms in Chilean university students. The study design was cross-sectional. A total of 934 first-year students at a Chilean public university completed a self-report questionnaire. To assess adherence to Mediterranean diet, an index validated in Chile (Chilean-MDI) was used, and depression, anxiety, and stress symptoms were assessed using the *Depression Anxiety and Stress Scale* (DASS-21). Logistic regression models were used to analyze the association of adherence to Mediterranean diet and its food groups with depression, anxiety, and stress symptoms adjusted for the main confounders. Students with moderate and high adherence to Mediterranean diet showed lower odds of depression [DASS-21 > 5, odds ratio (OR) = 0.64; 95% confidence interval (95%CI): 0.47-0.88] than those with low adherence to Mediterranean diet. The consumption of 1-2 servings/day of vegetables (OR = 0.63; 95%CI: 0.43-0.92), > 2 servings/week of nuts (OR = 0.41; 95%CI: 0.21-0.80), 1-2 servings/day of fruits (OR = 0.60; 95%CI: 0.42-0.85), 1-2 servings/week of fish and seafood (OR = 0.67; 95%CI: 0.48-0.94), and 1/2-3 units/week of avocado (OR = 0.67; 95%CI: 0.48-0.93) showed low odds of depressive symptoms. The consumption of whole grains and cereals (> 2 servings/day) (OR = 1.63; 95%CI: 1.02-2.61) showed the opposite association. Adherence to Mediterranean diet and consumption of fruits, vegetables, nuts, avocado, fish, and seafood are associated with a lower likelihood of depression in Chilean university students. New policies and educational strategies are recommended to improve diet quality and the mental health of the entire university community.

## Resumen

El objetivo de este estudio fue determinar la asociación entre la adherencia a la dieta mediterránea y sus grupos de alimentos y los síntomas depresivos en estudiantes universitarios chilenos. El diseño del estudio fue el transversal. Un total de 934 estudiantes de primer año de una universidad pública chilena completaron una encuesta de autoevaluación sobre la adherencia a la DM -utilizando un índice validado en Chile (IDM chileno)- y los síntomas de depresión, ansiedad y estrés, mediante el uso de la *Escala de Depresión, Ansiedad y Estrés* (DASS-21). Se utilizaron los modelos de regresión logística para analizar la asociación entre la adherencia a la dieta mediterránea y sus grupos alimenticios y los síntomas de depresión, ansiedad y estrés, ajustados por los principales factores de confusión. Los estudiantes con moderada o alta adherencia a la dieta mediterránea tenían menos probabilidades de presentar depresión [DASS-21 > 5, *odds ratio* (OR) = 0,64; intervalo de 95% de confianza (IC95%): 0,47-0,88] que aquellos con baja adherencia a la dieta mediterránea. Un consumo de 1-2 porciones por día de verduras (OR = 0,63; IC95%: 0,43-0,92), > 2 porciones por semana de nueces (OR = 0,41; IC95%: 0,21-0,80), 1-2 porciones por día de frutas (OR = 0,60; IC95%: 0,42-0,85), 1-2 porciones por semana de pescado y marisco (OR = 0,67; IC95%: 0,48-0,94) y 1/2-3 unidades por semana de aguacate (OR = 0,67; IC95%: 0,48-0,93) resultó en bajas probabilidades de síntomas depresivos. El consumo de cereales integrales (> 2 porciones por día) (OR = 1,63; IC95%: 1,02-2,61) tuvo una asociación opuesta. La adhesión a la dieta mediterránea y el consumo de frutas, verduras, nueces, aguacate, pescados y mariscos se asocian con una menor probabilidad de depresión en los estudiantes universitarios chilenos. Se sugiere la adopción de nuevas políticas y estrategias educativas para mejorar la calidad de la alimentación y promover la salud mental de toda la comunidad universitaria.

## Resumo

O objetivo do estudo foi determinar a associação da adesão à dieta mediterrânea e seus grupos alimentares com sintomas depressivos em estudantes universitários chilenos. O desenho do estudo foi transversal. Um total de 934 estudantes do primeiro ano de uma universidade pública chilena responderam a uma pesquisa de autoavaliação para analisar a adesão à dieta mediterrânea - por meio de um índice validado no Chile (IDM-chileno) - e sintomas de depressão, ansiedade e estresse, utilizando a *Escala de Depressão, Ansiedade e Estresse* (DASS-21). Modelos de regressão logística foram utilizados para examinar a associação entre a adesão à dieta mediterrânea e seus grupos alimentares com sintomas de depressão, ansiedade e estresse, ajustados para os principais fatores de confusão. Estudantes com adesão moderada ou alta à dieta mediterrânea apresentaram chances menores de ter depressão [DASS-21 > 5, *odds ratio* (OR) = 0,64; intervalo de 95% de confiança (IC95%): 0,47-0,88] do que aqueles com baixa adesão à dieta mediterrânea. Um consumo de 1-2 porções por dia de verduras (OR = 0,63; IC95%: 0,43-0,92), > 2 porções por semana de nozes (OR = 0,41; IC95%: 0,21-0,80), 1-2 porções por dia de frutas (OR = 0,60; IC95%: 0,42-0,85), 1-2 porções por semana de peixes e frutos do mar (OR = 0,67; IC95%: 0,48-0,94) e 1/2-3 unidades por semana de abacate (OR = 0,67; IC95%: 0,48-0,93) resultou em chances baixas de sintomas depressivos. O consumo de cereais integrais (> 2 porções por dia) (OR = 1,63; IC95%: 1,02-2,61) resultou na associação oposta. A adesão à dieta mediterrânea e o consumo de frutas, verduras, nozes, abacate, peixes e frutos do mar estão associados a uma menor probabilidade de depressão em estudantes universitários chilenos. Recomendamos a adoção de novas políticas e estratégias educacionais para melhorar a qualidade da alimentação e promover a saúde mental de toda a comunidade universitária.

## Introduction

Mental health disorders are among the leading causes of disability and the global health-related burden. The Global Burden of Diseases, Injuries, and Risk Factors (GBD) Study 2019 showed that the two most disabling mental disorders are depression and anxiety. A recent study conducted in 204 countries during the COVID-19 pandemic identified a global increase of 27.6% and 25.6% in cases of depressive and anxiety disorders, respectively, especially among women and younger populations ^1^.

The etiology of mental disorders includes biological, genetic, environmental, and lifestyle factors, such as diet ^2^. Many epidemiological studies have evaluated the association between individual food groups (i.e., fish, fruits, nuts, and vegetables) ^3^ ^,^ ^4^ ^,^ ^5^ ^,^ ^6^ and bioactive compounds (i.e., omega-3 fatty acids, folic acid, polyphenols, flavonoids, or B vitamins) ^7^ ^,^ ^8^ and mental health ^9^ ^,^ ^10^ ^,^ ^11^ ^,^ ^12^. However, diet is a multidimensional exposure that includes synergistic effects between different dietary components. Therefore, assessing dietary patterns is a more comprehensive approach to find diet-disease relationships and provide public health recommendations.

The Mediterranean diet is a dietary pattern based on the healthy traditional eating habits of the populations from the countries around the Mediterranean Sea, which includes a high consumption of plant-based foods (i.e., fruits, vegetables, whole grains, nuts, and seeds, and legumes and pulses), fish, and unsaturated fats (i.e., such as olive oil), a moderate consumption of dairy products and eggs, and a low consumption of red meat and processed meat products ^13^ ^,^ ^14^. Some of its dietary components have been shown to play a key role in depression. Thus, the intake of n-3 polyunsaturated fatty acids (n-3 PUFAs) [docosahexaenoic acid (DHA), eicosapentaenoic acid (EPA), and alpha-linoleic acid (ALA)], which are present in foods such as fish and vegetable oils, have a beneficial effect on depression. One hypothesis suggests that n-3 PUFAs affect depressive symptoms by several possible pathways, such as anti-inflammatory effects, neuroendocrine modulation, and neuroprotective/neurotrophic mechanisms ^15^ ^,^ ^16^ ^,^ ^17^. Some population-based studies have also reported an inverse association between adherence to Mediterranean diet and depressive disorder, but their results are contradictory ^18^ ^,^ ^19^ ^,^ ^20^. Although a meta-analysis of cohort studies found a nonsignificant association between adherence to Mediterranean diet and risk of depression ^18^, recent population-based prospective studies suggest lower odds of depression in individuals with high adherence to Mediterranean diet ^21^. Therefore, further studies are needed to clarify and strengthen the evidence regarding the association between this dietary pattern and mental health disorders.

The transition to university is a critical period for young adults, in which attending university can be a stressful time for most students. Besides academic pressure, some students have to handle several family and work responsibilities, while others face the stressful task of separating from their family and hometown. Thus, many students experience the onset of mental health disorders during the first years at university ^22^. On the other hand, these first years are their first opportunity to make their own food choices ^23^. Poor cooking skills, obesogenic environments with high availability of fast food, lack of time, and financial worries can lead to unhealthy eating habits and sedentary patterns, promoting weight gain, which may persist into adulthood ^24^ ^,^ ^25^.

In Chile, no previous study has analyzed the relationship between adherence to Mediterranean diet and mental health disorders in this highly vulnerable population. Therefore, this study aimed to assess: (1) the association between adherence to Mediterranean diet and depression, anxiety, and stress symptoms; and (2) the relationship between the different Mediterranean diet food groups and depressive disorders in a sample of Chilean university students during their first year at the university.

## Material and methods

### Study design and participants

**Figure 1:**
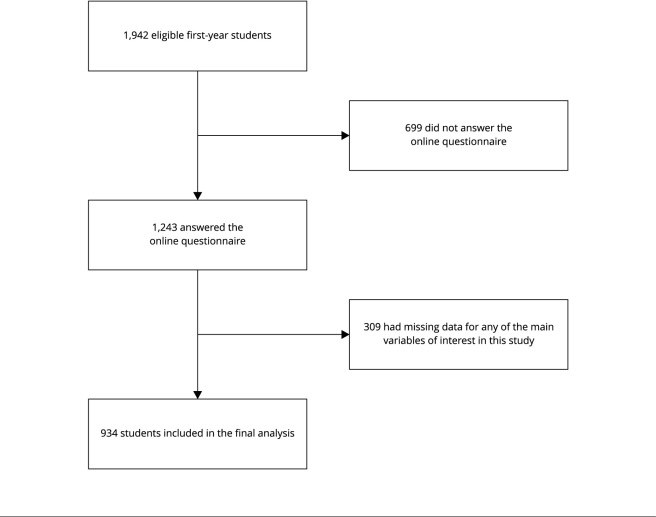
Flowchart of the study participants.

This cross-sectional online study recruited participants in March 2021. All first-year students at a Chilean university were considered eligible and invited to participate in the study. Of the 1,942 eligible students invited to participate, 1,243 (63.5%) agreed to answer an online questionnaire, of whom 934 (48.1%) provided complete data for the main variables of interest in the study (Figure 1).

Supplementary Material (Table S1. Supplementary Material https://cadernos.ensp.fiocruz.br/static//arquivo/supl-e00206722_8682.pdf) shows a comparison of the descriptive characteristics of the total number of university students initially selected and the final sample. Generally, no substantial differences were found, especially regarding the main variables of the study (i.e., depression, anxiety, and stress symptoms and adherence to Mediterranean diet).

### Procedures

Participants were invited to voluntarily participate in an online health survey. After providing informed consent electronically, participants completed a 20-minute online questionnaire. All students who signed the informed consent form and completed the questionnaire were considered in the analyses.

### Measurements

#### Assessment of adherence to the Mediterranean diet

A Mediterranean dietary index validated in Chile (Chilean-MDI) was applied. In total, 14 food groups were assessed [fruits, vegetables, legumes, nuts, fish and seafood, whole grains and cereals, fat-free or fermented dairy products, full-fat dairy products, lean meat, red meat and processed meat, wine, olive oil, other healthy fats (canola), and avocado and sugars]. The maximum score was 14 points. Higher scores represent greater adherence to Mediterranean diet. The following categories are considered: high (9-14 points), moderate (5-8.5 points), and low (< 5 points) adherence ^26^. For this study, the high and moderate categories were grouped (> 5 points).

#### Assessment of depression, anxiety, and stress symptoms

Depression, anxiety, and stress symptoms were assessed using the *Depression Anxiety and Stress Scale* (DASS-21). This tool was validated in Chile ^27^ and has good reliability and validity in Hispano-American, British, and Australian adults.

Students rated 21 statements, seven for each construct assessed (depression, anxiety, and stress symptoms), answering to what extent the statement applied to them over the past week, on a four-point scale (0-3). The answers ranged from “did not apply to me at all” to “applied to me very much or most of the time” (lower scores corresponded to reduced symptoms). Thus, each construct has a total score ranging from 0 to 21 points. A score > 5 points was the cut-off point for depression and stress symptoms. A score > 4 points was the cut-off point for anxiety symptoms ^28^.

#### Other covariables

This study considered other variables of interest:

(a) Sociodemographic data: age (years), sex (male, female), maternal schooling level (secondary education or lower, or higher education);

(b) Body mass index (BMI): estimated from self-reported weight and height. The cut-off points were < 18.5kg/m^2^ for underweight, 18.5-24.9kg/m^2^ for normal weight, 25-29.9kg/m^2^ for overweight, and ≥ 30kg/m^2^ for obesity;

(c) Sedentary lifestyle: according to the following question of the *Physical Activity Questionnaire Short Form* (IPAQ-SF): “During the last 7 days, how much time did you spend sitting on a week day?” ^29^ ^,^ ^30^. Students who spent ≥ 8 hours/day sitting were considered sedentary;

(d) Physical activity: assessed using the IPAQ-SF. Students were grouped into two categories (yes or no) according to their compliance with the recommendation of at least five days of moderate physical activity and walking ^31^;

(e) Tobacco use: assessed by the *Alcohol, Smoking and Substance Involvement Screening Test* (ASSIST). “In the past three months, how often have you used tobacco products?” (never, once or twice, monthly, weekly, or daily or almost daily) ^32^. The weekly and daily categories were grouped;

(f) Alcohol consumption was assessed using the same ASSIST question: “In the past three months, how often have you used alcoholic beverages?” (never, once or twice, monthly, weekly, or daily or almost daily) ^32^. The weekly and daily categories were grouped.

### Statistical analysis

Data are presented as mean and standard deviation (SD) for continuous variables and number and percentages for categorical variables. Student’s t-test and the Mann-Whitney test were used for variables with a normal or non-normal distribution, respectively. The chi-squared test was used for categorical variables. Logistic regression models were used to analyze the association between adherence to Mediterranean diet and depression, anxiety, and stress symptoms. Mediterranean diet food groups were also analyzed. In this analysis, consumption below recommended levels was divided into two or more categories for a better analysis of the isolated effects of each food group. Results are presented as odds ratios (ORs) and 95% confidence intervals (95%CIs). Models were adjusted for variables associated with Mediterranean diet and depressive symptoms ^33^ ^,^ ^34^ ^,^ ^35^ ^,^ ^36^ ^,^ ^37^, such as sex (male, female), BMI (underweight, normal weight, overweight, or obesity), maternal schooling level (secondary education or lower, or higher education), tobacco use (never, occasionally, or weekly/daily), alcohol use (never, occasionally, or weekly/daily), sedentary lifestyle (yes or no), and physical activity (compliance with the recommendation: yes or no). Due to the evidence of interaction of the sex variable with depression and diet ^36^, the Mantel-Haenszel homogeneity test was used. Moreover, considering the low frequency of wine consumption, a sensibility analysis was performed for the total Chilean-MDI score and the score without “wine” (Supplementary Material. Table S2. Supplementary Material https://cadernos.ensp.fiocruz.br/static//arquivo/supl-e00206722_8682.pdf). All statistical analyses were performed using Stata version 15 (https://www.stata.com). The sample size for the a priori and post hoc analyses was determined using G*Power, version 3.1.9.6 (http://www.psycho.uni-duesseldorf.de/abteilungen/aap/gpower3). To estimate the a priori sample size, this study considered a medium effect size (1.3), a power of 80%, a two-tailed model, H0 = 0.20 for Pr(Y = 1 | X = 1), and α = 0.05. The estimated sample size was 721 participants. To estimate the post hoc power for each analysis, this study used the sample size, a two-tailed model, H0 = 0.20 for Pr(Y = 1 | X = 1), and α = 0.05 ^38^.

### Ethical approval

This study was approved by the Research Ethics Committee of the La Frontera University (Chile; protocol n. 150/21).

## Results

Of the 1,942 eligible students invited to participate, 1,243 (63.5%) agreed to answer an online questionnaire and 934 provided complete data for the main variables of interest of this study (Figure 1). Table 1 presents the main characteristics of the participants according to sex. A total of 494 (52.9%) were females, with a mean age of 19.2 years old (SD = 1.6), and 67% reported that their mother’s schooling level was secondary education or lower. More than half of students were of normal weight. Regarding lifestyle habits, most students practiced physical activity five or more times per week (70.1%), were not sedentary (72.1%), did not smoke (79.2%), and consumed alcohol occasionally (60.4%). Regarding mental disorders according to the DASS-21, all students reported a high prevalence of depression (23.9%), anxiety (29.4%), and stress (37.9%) symptoms, with a higher prevalence in women. Finally, adherence to Mediterranean diet was high (60.8%).

**Table 1:** Sociodemographic and lifestyle characteristics of Chilean first-year university students according to sex.

Characteristics	Total	Female	Male	p-value
Participants [n (%)]	934 (100.0)	494 (52.9)	440 (47.1)	
Age [median (p25-p75)]	19.0 (18-19)	19.0 (18-19)	19.0 (18-19)	0.6455
Maternal schooling level [n (%)]				0.989
Secondary education or lower	626 (67.0)	331 (67.0)	295 (67.1)	
Higher education	308 (33.0)	163 (33.0)	145 (32.9)	
BMI (kg/m^2^) [n (%)]				0.592
Underweight	40 (4.3)	17 (3.5)	23 (5.2)	
Normal weight	550 (58.9)	292 (59.1)	258 (58.6)	
Overweight	251 (26.8)	134 (27.1)	117 (26.6)	
Obesity	93 (10.0)	51 (10.3)	42 (9.6)	
Sedentary lifestyle [n (%)]				0.078
Yes (≥ 8 hours/day sitting)	261 (27.9)	126(25.5)	135 (30.7)	
No (< 8 hours/day sitting)	673 (72.1)	368 (74.5)	305 (69.3)	
Physical activity [n (%)] *				< 0.0001
Yes	655 (70.1)	318 (64.4)	337 (76.6)	
No	279 (29.9)	176 (35.6)	103 (23.4)	
Tobacco use [n (%)]				0.844
Never	740 (79.2)	394 (79.8)	346 (78.6)	
Occasionally	151 (16.2)	79 (16.0)	72 (16.4)	
Weekly or daily	43 (4.6)	21 (4.2)	22 (5.0)	
Alcohol use [n (%)]				0.186
Never	311 (33.3)	172 (34.8)	139 (31.6)	
Occasionally	564 (60.4)	297 (60.1)	267 (60.7)	
Weekly or daily	59 (6.3)	25 (5.1)	34 (7.7)	
DASS-21 score [median (p25-p75)] **				
Depression	2 (1-5)	3 (1-6)	2 (0-4)	< 0.0001
Anxiety	2 (1-5)	3 (1-7)	2 (0-4)	< 0.0001
Stress	4 (2-7)	5 (2-8)	4 (1-6)	< 0.0001
DASS-21 symptoms [n (%)] ***				
Depression (> 5 points)	223 (23.9)	139 (28.1)	84 (19.1)	0.001
Anxiety (> 4 points)	275 (29.4)	190 (38.5)	85 (19.3)	< 0.0001
Stress (> 5 points)	354 (37.9)	219 (44.3)	135 (30.7)	< 0.0001
Mediterranean diet score [mean ± SD] ^#^	5.2 ± 1.6	5.4 ± 1.6	5.1 ± 1.5	0.0021
Adherence to Meditteranean diet [n (%)] ^#^				0.249
High/Moderate (5-14 points)	568 (60.8)	309 (62.5)	259 (58.9)	
Low (< 5 points)	366 (39.2)	185 (37.5)	181 (41.1)	

BMI: body mass index; DASS-21: *Depression Anxiety and Stress Scale*; SD: standard deviation.

Note: data are presented as mean ± SD, median (p25-p75), or number (%). Student’s t-test and the Mann-Whitney test were used for variables with normal or non-normal distributions, respectively. The χ^2^ test was used for categorical variables.

* Physical activity according to compliance with the recommendation of at least five days of moderate activity and/or walking ^31^;

** DASS-21 proposed by Mella et al. ^27^;

*** DASS-21 symptoms according to the cut-off points proposed by Román et al. ^28^;

^#^ Mediterranean diet adapted to Chile, according to the score proposed by Echeverría et al. ^26^.

Compliance with Mediterranean diet recommendations was low, especially for the following food groups: wine (0.4%), fish and seafood (3.8%), olive oil (4.3%), avocado (4.6%), vegetables (6.8%), canola oil (6.9%), nuts (9.3%), fruits (12.9%), and whole grains (13.4%). Regarding both the healthy and unhealthy food groups (fats, processed meat, full-fat dairy products, and sweetened beverages), females complied with the recommendations to a greater extent than males (Table 2).

**Table 2:** Food consumption according to the recommendations of the Mediterranean diet, by sex, in Chilean first-year university students.

Food groups	Recommendation	Total	Female	Male	p-value
		n (%)	n (%)	n (%)	
Vegetables	3 servings/day	63 (6.8)	38 (7.7)	25 (5.7)	0.109
Legumes	> 2 servings/week	90 (9.6)	41 (8.3)	49 (11.1)	0.051
Nuts	> 2 servings/week	87 (9.3)	46 (9.3)	41 (9.3)	0.928
Fruits	≥ 2 servings/day	120 (12.9)	64 (13.0)	56 (12.7)	0.913
Whole grains and cereals	≥ 2 servings/day	125 (13.4)	67 (13.6)	58 (13.2)	0.039
Lean meat	5-8 servings/week	95 (10.2)	45 (9.1)	50 (11.4)	0.005
Fish and seafood	> 2 servings/week	35 (3.8)	17 (3.4)	18 (4.1)	0.122
Fats and processed meat	< 1 serving/week	302 (32.3)	205 (41.5)	97 (22.1)	< 0.001
Full-fat dairy products	< 1serving /day	480 (51.4)	277 (56.1)	203 (46.1)	0.002
Fat-free and fermented dairy products	> 1 serving/day	237 (25.4)	127 (25.7)	110 (23.7)	0.651
Olive oil	≥ 3 teaspoons/day	40 (4.3)	29 (5.9)	11 (2.5)	0.022
Other healthy fats					
Avocado	≥ 3 units/week	43 (4.6)	21 (4.3)	22 (5.0)	0.180
Canola oil	Regular consumption	64 (6.9)	36 (7.3)	28 (6.4)	0.147
Sugars					
Sugar	< 4 teaspoons/day	412 (44.1)	212 (42.9)	200 (45.5)	0.001
Sweets	No consumption	501 (53.6)	264 (53.4)	237 (53.8)	0.897
Sweetened beverages	No consumption	507 (54.3)	308 (62.4)	199 (45.2)	< 0.001
Wine	1-2 glasses ≥ 4 days/week	4 (0.4)	3 (0.3)	1 (0.2)	0.945

Table 3 presents the association between depressive symptoms and adherence to Mediterranean diet according to the index adjusted for the main confounders. The model shows that, compared with students with low adherence, students with moderate and high adherence to Mediterranean diet have a lower risk of depression (OR = 0.64; 95%CI: 0.47-0.88; power 99%). The results did not vary substantially between females and males. We conducted an interaction analysis for the sex variable using the Mantel-Haenszel homogeneity test and it was not significant (Pr > χ^2^ = 0.9649). On the other hand, we found no association for anxiety or stress symptoms, which had a power of 86% and 56%, respectively (Table 3).

**Table 3:** Association between depression, anxiety, and stress symptoms and adherence to the Mediterranean diet in Chilean first-year university students according to the Mediterranean dietary index validated in Chile (Chilean-MDI).

Chilean-MDI	Depression			Anxiety			Stress		
	OR	95%CI	p-value	OR	95%CI	p-value	OR	95%CI	p-value
Low adherence	1.00	-	-	1.00	-	-	1.00	-	-
High/Moderate adherence	0.64	0.47-0.88	0.006	0.78	0.57-1.05	0.10	0.84	0.63-1.11	0.22

95%CI: 95% confidence interval; OR: odds ratio.

Note: model adjusted by sex (female, male), age (years old), maternal schooling level (secondary education or lower, or higher education), body mass index (underweight, normal weight, overweight, or obesity), tobacco use (never, occasionally, or weekly/daily), alcohol use (never, occasionally, or weekly/daily), sedentary lifestyle (yes or no), and physical activity (compliance with the recommendation: yes or no).

In the fully adjusted models, the OR (95%CI) for students with a high consumption of the recommended food groups showed a low risk of depressive symptoms: 0.63 (95%CI: 0.43-0.92) for 1-2 servings per day of vegetables, 0.41 (95%CI: 0.21-0.80) for > 2 servings per week of nuts, 0.60 (95%CI: 0.42-0.85) for 1-2 servings per day of fruits, 0.67 (95%CI: 0.48-0.94) for 1-2 servings per week of fish and seafood, and 0.67 (95%CI: 0.48-0.93) for 1/2-3 units per week of avocado. The consumption of whole grains and cereals showed the opposite association, since students who consumed more than two servings per day had a higher risk of depressive symptoms: 1.63 (1.02-2.61). The association was statistically significant for all food groups (Table 4).

**Table 4:** Association between depression symptoms and food groups in Chilean first-year university students.

Food groups	OR (95%CI)
Vegetables (servings/day)	
< 1	1.00
1-2	0.63 (0.43-0.92)
> 2	0.78 (0.40-1.51)
Per week	92%
Legumes (servings/week)	
< 1	1.00
1-2	0.86 (0.58-1.27)
> 2	1.07 (0.59-1.95)
Per week	15%
Nuts (servings/week)	
< 1	1.00
1-2	1.24 (0.87-1.78)
> 2	0.41 (0.21-0.80) *
Per week	83%
Fruits (servings/day)	
< 1	1.00
1-2	0.60 (0.42-0.85) *
> 2	0.73 (0.43-1.23)
Per week	98%
Whole grains cereals (servings/day)	
< 1	1.00
1-2	0.88 (0.62-1.24)
> 2	1.63 (1.02-2.61) *
Per week	42%
Lean meat and poultry (servings/week)	
< 1	1.00
1-4	0.68 (0.44-1.06)
5-8	0.82 (0.44-1.06)
> 8	0.93 (0.22-3.88)
Per week	17%
Fish and seafood (servings/week)	
< 1	1.00
1-2	0.67 (0.48-0.94) *
> 2	0.66 (0.27-1.61)
Per week	99%
Fatty and processed meat (servings/week)	
< 1	1.00
1-2	1.08 (0.75-1.55)
> 2	1.27 (0.82-1.95)
Per week	18%
Whole fat dairy products (servings/day)	
< 1	1.00
≤ 1	0.87 (0.54-1.41)
Per week	48%
Low fat dairy products (servings/day)	
0	1.00
1	0.50 (0.16-1.47)
> 1	0.57 (0.18-1.74)
Per week	100%
Olive oil (teaspoons/day)	
< 1	1.00 (Reference)
1-2	1.27 (0.45-3.62)
> 2	0.98 (0.27-3.51)
Per week	6%
Avocado (unit/week)	
0-1/2	1.00 (Reference)
1/2-3	0.67 (0.48-0.93) *
> 3	1.01 (0.50-2.08)
Per week	5%
Sugar (teaspoons/day)	
< 4	1.00 (Reference)
1-4	1.29 (0.85-1.97)
0	0.86 (0.61-1.23)
Per week	54%

Note: model adjusted by sex (female, male), age (years old), maternal schooling level (secondary education or lower, or higher education), body mass index - BMI (underweight, normal weight, overweight, or obesity), tobacco use (never, occasionally, or weekly/daily), alcohol use (never, occasionally, or weekly/daily), sedentary lifestyle (yes or no), and physical activity (compliance with the recommendation: yes or no). Wine was not included as a food group, since n = 13 participants.

* p < 0.05.

## Discussion

Our data showed that Chilean university students with greater adherence (high or moderate) to Mediterranean diet were 36% less likely to have depressive symptoms compared with students with low adherence in the fully adjusted models. Moreover, higher consumption of some food groups recommended for Mediterranean diet (vegetables, nuts, fruits, fish and seafood, and avocado) was associated with lower depressive symptoms.

Dietary intake and patterns change throughout life and are influenced by biological ^39^ and social ^40^ ^,^ ^41^ issues and university entrance ^42^ ^,^ ^43^. In this sense, sex seems to be an important feature, as males and females have different biological food consumption needs, which directly and indirectly promotes the adoption of different eating habits ^44^. Moreover, sex can determine biological changes that increase the likelihood of mental disorders in females, with a possible increase in incidence during the COVID-19 pandemic ^45^. These mechanisms justify the greater adherence to Mediterranean diet and the frequency of depression in women, as shown in this study. However, in contrast to studies that found an association only in women ^46^ ^,^ ^47^, in this study, the association was in the entire population (i.e., in males and females), and we observed no interaction between sex, greater adherence to Mediterranean diet, and lower depressive symptoms. Thus, sex was a control in the adjusted models, and this variable was not subjected to stratification analysis.

Regarding food consumption patterns, the frequency of adequate consumption of some foods was low compared with other studies involving university students, especially when in Mediterranean countries or countries closer to the culture, such as vegetables (6.8% versus 18.1% to 45,5%) ^48^ ^,^ ^49^, fruits (12.9% versus 23.9%) ^49^, nuts (9.3% versus 66.9%) ^49^, and fish and seafood (3.8% versus 41% to 77.3%) ^49^ ^,^ ^50^. Adequate consumption of the following healthy food has been associated with a lower prevalence of depressive symptoms: fruits and vegetables ^51^, due to the presence of vitamins and folates that can act as antioxidants in the process of regeneration and production of neurotransmitters ^52^ ^,^ ^53^; fish and seafood ^54^ ^,^ ^55^ and avocado, due to their anti-inflammatory unsaturated fatty acids; and greater consumption of nuts ^5^, due to the modulation of neuronal factors ^52^ ^,^ ^53^.

On the other hand, a higher consumption (> 2 servings per day) of whole grains and cereals showed a greater likelihood of depressive symptoms. Although the literature presents the consumption of whole grains and cereals as a protective factor for depression ^52^ ^,^ ^53^, the consumption of refined cereals may not be beneficial ^56^ and may also be associated with a higher frequency of depressive symptoms ^57^. We could hypothesize that students probably overreported whole grain consumption due to the inclusion of breakfast cereals in this group, which are mostly ultra-processed food and have a complex formulation, including ingredients such as glucose syrups, invert sugar, sweeteners, and additives, besides neo-formed contaminants harmful to health (e.g., acrylamide) ^58^. These findings, along with the differences in the frequency of consumption of important food groups (vegetables, fruits, nuts, and fish and seafood), reinforce the importance of a broader dietary assessment for this population, recognizing cultural differences and the impossibility of comparing consumption frequencies, especially from different cultures ^59^.

The consumption of fruits ^51^ ^,^ ^57^ and nuts ^5^ is consistently associated with a reduction in depressive symptoms. However, the effect of consuming isolated foods has limitations of interpretation ^51^ ^,^ ^53^ ^,^ ^57^. This would justify the lack of association in our study for the consumption of fruits, vegetables, avocado, and fish and seafood. Similarly, different foods and nutrients have a synergistic effect on each other, which makes dietary pattern analysis recommended ^60^. However, this reinforces the importance of a broader analysis of food consumption, such as the analysis of adherence to Mediterranean diet using Chilean-MDI.

This study found that high and moderate adherence to Mediterranean diet leads to a lower risk of depressive symptoms. This can be attributed to most micronutrients, including adequate B vitamins and folate and antioxidant compounds existing in Mediterranean diet, allowing simultaneous modulation between different body systems. On the other hand, different compounds have been associated with depressive symptoms, such as inflammatory cytokines, interleukin-6, tumor necrosis factor-α, and C-reactive protein. The main mechanisms attributed to Mediterranean diet can be explained by the modulation of glucocorticoids, promotion of neurogenesis, decrease in oxidative stress markers, reduction of inflammatory markers, and modulation of the microbiota and epigenetic state ^52^ ^,^ ^53^. These mechanisms have a unidirectional character, starting from Mediterranean diet and resulting in depressive symptoms. Since longitudinal epidemiological studies corroborate this directionality ^61^ ^,^ ^62^ ^,^ ^63^ and the literature does not support the effect in the opposite direction ^64^, we reinforce the directionality identified in this study. Finally, another mechanism that may help understand this association is that greater adherence to Mediterranean diet is associated with lower consumption of ultra-processed foods ^65^, which in turn are associated with a greater presence of depressive symptoms ^66^.

Greater adherence to Mediterranean diet was associated with lower odds of anxiety symptoms. This association has been reported in populations of adults and university students ^57^ ^,^ ^67^. On the other hand, the association between Mediterranean diet and stress is not a consensus in the available evidence. Some studies show a lower risk of stress in students with moderate/high adherence to Mediterranean diet ^57^ ^,^ ^68^, while another study found no association with general stress ^69^.

As a limitation of this study, we highlight the impossibility of assessing causality (directionality or bidirectionality) due to the cross-sectional design. Another limitation is that all data were self-reported, using an online questionnaire; thus, the answers may be affected by recall and social desirability bias. Although self-reported weight and height are a limitation of this study, the literature shows evidence of good agreement between self-reported and measured values in young adults, which minimizes the bias of the BMI variable ^70^ ^,^ ^71^. Regarding sample size, we provided data for less than 50% of all eligible students, which may cause biases (since the participating students may be different from those who did not participate in terms of exposure and outcome variables). Thus, our results should be interpreted with caution. As strengths, we highlight the use of validated tools to assess Mediterranean diet and depression, anxiety, and stress symptoms, the use of models adjusted for confounders, and high power for most of the associations found.

## Conclusion

Students who adhered to Mediterranean diet (highly or moderately) were 36% less likely to have depressive symptoms compared with students with lower adherence in the fully adjusted models. Moreover, higher consumption of vegetables, nuts, fruits, fish and seafood, and avocado was associated with lower depressive symptoms. Mediterranean diet was not associated with anxiety and stress symptoms.

New policies and educational strategies are recommended to improve diet quality and the mental health of the entire university community.

## References

[1] GBD 2019 Diseases and Injuries Collaborators (2020). Global burden of 369 diseases and injuries in 204 countries and
territories, 1990-2019: a systematic analysis for the Global Burden of
Disease Study 2019. Lancet.

[2] Saveanu RV, Nemeroff CB (2012). Etiology of depression genetic and environmental
factors. Psychiatr Clin North Am.

[3] Yoshikawa E, Nishi D, Matsuoka Y (2015). Fish consumption and resilience to depression in Japanese company
workers a cross-sectional study. Lipids Health Dis.

[4] Liu X, Yan Y, Li F, Zhang D (2016). Fruit and vegetable consumption and the risk of depression a
meta-analysis. Nutrition.

[5] Fernández-Rodríguez R, Jiménez-López E, Garrido-Miguel M, Martínez-Ortega IA, Martínez-Vizcaíno V, Mesas AE (2022). Does the evidence support a relationship between higher levels of
nut consumption, lower risk of depression, and better mood state in the
general population A systematic review. Nutr Rev.

[6] Cobo-Cuenca AI, Garrido-Miguel M, Soriano-Cano A, Ferri-Morales A, Martínez-Vizcaíno V, Martín-Espinosa NM (2019). Adherence to the mediterranean diet and its association with body
composition and physical fitness in Spanish university
students. Nutrients.

[7] Jia S, Hou Y, Wang D, Zhao X (2022). Flavonoids for depression and anxiety: a systematic review and
meta-analysis.. Crit Rev Food Sci Nutr.

[8] Lin K, Li Y, Toit ED, Wendt L, Sun J (2021). Effects of polyphenol supplementations on improving depression,
anxiety, and quality of life in patients with depression. Front Psychiatry.

[9] Dana-Alamdari L, Kheirouri S, Noorazar SG (2015). Serum 25-hydroxyvitamin D in patients with major depressive
disorder. Iran J Public Health.

[10] Kim TH, Choi J-Y, Lee HH, Park Y (2015). Associations between dietary pattern and depression in Korean
adolescent girls.. J Pediatr Adolesc Gynecol.

[11] Sanhueza C, Ryan L, Foxcroft DR (2013). Diet and the risk of unipolar depression in adults systematic
review of cohort studies. J Hum Nutr Diet.

[12] Sánchez-Villegas A, Verberne L, De Irala J, Ruíz-Canela M, Toledo E, Serra-Majem L (2011). Dietary fat intake and the risk of depression the SUN
project. PLoS One.

[13] Willett EC, Sacks F, Trichopoulou A, Drescher G, Ferro-Luzzi A, Helsing E (1995). Mediterranean diet pyramid: a cultural model for healthy
eating.. Am J Clin Nutr.

[14] Willett WC (2006). The Mediterranean diet science and practice. Public Health Nutr.

[15] Grosso G, Micek A, Marventano S, Castellano S, Mistretta A, Pajak A (2016). Dietary n-3 PUFA, fish consumption and depression a systematic
review and meta-analysis of observational studies. J Affect Disord.

[16] Skarupski KA, Tangney C, Li H, Ouyang B, Evans DA, Morris MC (2010). Longitudinal association of vitamin B-6, folate, and vitamin B-12
with depressive symptoms among older adults over time. Am J Clin Nutr.

[17] Xu Y, Wang C, Klabnik JJ, O'Donnell JM (2014). Novel therapeutic targets in depression and anxiety: antioxidants
as a candidate treatment.. Curr Neuropharmacol.

[18] Shafiei F, Salari-Moghaddam A, Larijani B, Esmaillzadeh A (2019). Adherence to the mediterranean diet and risk of depression a
systematic review and updated meta-analysis of observational
studies. Nutr Rev.

[19] Sánchez-Villegas A, Martínez-González MA, Estruch R, Salas-Salvadó J, Corella D, Covas MI (2013). Mediterranean dietary pattern and depression the PREDIMED
randomized trial. BMC Med.

[20] Yin W, Löf M, Chen R, Hultman CM, Fang F, Sandin S (2021). Mediterranean diet and depression a population-based cohort
study. Int J Behav Nutr Phys Act.

[21] Oddo VM, Welke L, McLeod A, Pezley L, Xia Y, Maki P (2022). Adherence to a mediterranean diet is associated with lower
depressive symptoms among U S. adults. Nutrients.

[22] Pedrelli P, Nyer M, Yeung A, Zulauf C, Wilens T (2015). Buku panduan program ijazah dasar sesi akademik
2013/2014. Acad Psychiatry.

[23] Deshpande S, Basil MD, Basil DZ (2009). Factors influencing healthy eating habits among college students
an application of the health belief model. Health Mark Q.

[24] Payne-Sturges DC, Tjaden A, Caldeira KM, Vincent KB, Arria AM (2018). Student hunger on campus food insecurity among college students
and implications for academic institutions. Am J Health Promot.

[25] Vella-Zarb RA, Elgar FJ (2009). The "freshman 5" a meta-analysis of weight gain in the freshman
year of college. J Am Coll Health.

[26] Echeverría G Urquiaga I.Concha MJ.Dussaillant C.Villarroel L.Velasco
N (2016). Validación de cuestionario autoaplicable para un índice de
alimentación mediterránea en Chile. Rev Méd Chile.

[27] Mella FR, Vinet EV, Muñoz AMA (2014). Escalas de Depresión, Ansiedad y Estrés (DASS-21) adaptación y
propiedades psicométricas en estudiantes secundarios de
Temuco. Rev Argent Clín Psicol.

[28] Román F, Santibañez P, Vinet EV (2016). Uso de Escalas de Depresión, Ansiedad, Estrés (DASS-21) como
instrumento de tamizaje en jóvenes con problemas clínicos. Acta Investigación Psicol.

[29] Lee PH, Macfarlane DJ, Lam T, Stewart SM (2011). Validity of the International Physical Activity Questionnaire
Short Form (IPAQ-SF) a systematic review. Int J Behav Nutr Phys Act.

[30] Palma-Leal X, Costa-Rodríguez C, Barranco-Ruiz Y, Hernández-Jaña S, Rodríguez-Rodríguez F (2022). Fiabilidad del Cuestionario Internacional de Actividad Física
(IPAQ)-versión corta y del Cuestionario de Autoevaluación de la Condición
Física (IFIS) en estudiantes universitarios chilenos. Journal of Movement & Health.

[31] Crespo-Salgado JJ, Delgado-Martín JL, Blanco-Iglesias O, Aldecoa-Landesa S (2015). Guía básica de detección del sedentarismo y recomendaciones de
actividad física en atención primaria.. Aten Primaria.

[32] Group WAW (2002). The Alcohol, Smoking and Substance Involvement Screening Test
(ASSIST) development, reliability and feasibility. Addiction.

[33] Zaragoza-Martí A, Cabañero-Martínez MJ, Hurtado-Sánchez JA, Laguna-Pérez A, Ferrer-Cascales R (2018). Evaluation of Mediterranean diet adherence scores a systematic
review. BMJ Open.

[34] Kang M, Park S-Y, Shvetsov YB, Wilkens LR, Marchand LL, Boushey CJ (2019). Sex differences in sociodemographic and lifestyle factors
associated with diet quality in a multiethnic population.. Nutrition.

[35] Koutsonida M, Kanellopoulou A, Markozannes G, Gousia S, Doumas MT, Sigounas DE (2021). Adherence to Mediterranean diet and cognitive abilities in the
Greek Cohort of Epirus Health Study. Nutrients.

[36] Camilleri GM, Méjean C, Kesse-Guyot E, Andreeva VA, Bellisle F, Hercberg S (2014). The associations between emotional eating and consumption of
energy-dense snack foods are modified by sex and depressive
symptomatology. J Nutr.

[37] Gutiérrez-Rojas L, Porras-Segovia A, Dunne H, Andrade-González N, Cervilla JA (2020). Prevalence and correlates of major depressive disorder a
systematic review. Braz J Psychiatry.

[38] Faul F, Erdfelder E, Buchner A, Lang AG (2009). Statistical power analyses using G*Power 3 1: tests for
correlation and regression analyses. Behav Res Methods.

[39] Wakimoto P, Block G (2001). Dietary intake, dietary patterns, and changes with age: an
epidemiological perspective.. J Gerontol A Biol Sci Med Sci.

[40] Lee S, Cho E, Grodstein F, Kawachi I, Hu FB, Colditz GA (2005). Effects of marital transitions on changes in dietary and other
health behaviours in US women. Int J Epidemiol.

[41] Nasuti G, Blanchard C, Naylor P-J, Levy-Milne R, Warburton DER, Benoit C (2014). Comparison of the dietary intakes of new parents, second-time
parents, and nonparents: a longitudinal cohort study.. J Acad Nutr Diet.

[42] Hilger J, Loerbroks A, Diehl K (2017). Eating behaviour of university students in Germany dietary
intake, barriers to healthy eating and changes in eating behaviour since the
time of matriculation. Appetite.

[43] Bárbara R, Ferreira-Pêgo C (2020). Changes in eating habits among displaced and non-displaced
university students. Int J Environ Res Public Health.

[44] Li K-K, Concepcion RY, Lee H, Cardinal BJ, Ebbeck V, Woekel E (2012). An examination of sex differences in relation to the eating
habits and nutrient intakes of university students.. J Nutr Educ Behav.

[45] Xiong J, Lipsitz O, Nasri F, Lui LMW, Gill H, Phan L (2020). Impact of COVID-19 pandemic on mental health in the general
population a systematic review. J Affect Disord.

[46] Saneei P, Hajishafiee M, Keshteli AH, Afshar H, Esmaillzadeh A, Adibi P (2016). Adherence to Alternative Healthy Eating Index in relation to
depression and anxiety in Iranian adults. Br J Nutr.

[47] Rahmani J, Milajerdi A, Dorosty-Motlagh A (2018). Association of the Alternative Healthy Eating Index (AHEI-2010)
with depression, stress and anxiety among Iranian military
personnel. J R Army Med Corps.

[48] Rodrigues VM, Bray J, Fernandes AC, Bernardo GL, Hartwell H, Martinelli SS (2019). Vegetable consumption and factors associated with increased
intake among college students a scoping review of the last 10
years. Nutrients.

[49] Kyrkou C, Tsakoumaki F, Fotiou M, Dimitropoulou A, Symeonidou M, Menexes G (2018). Changing trends in nutritional behavior among university students
in Greece, between 2006 and 2016. Nutrients.

[50] El Ansari W, Suominen S, Samara A (2015). Eating habits and dietary intake is adherence to dietary
guidelines associated with importance of healthy eating among undergraduate
university students in Finland?. Cent Eur J Public Health.

[51] Dharmayani PNA, Juergens M, Allman-Farinelli M, Mihrshahi S (2021). Association between fruit and vegetable consumption and
depression symptoms in young people and adults aged 15-45 a systematic
review of cohort studies. Int J Environ Res Public Health.

[52] Pano O, Martínez-Lapiscina EH, Sayón-Orea C, Martinez-Gonzalez MA, Martinez JA, Sanchez-Villegas A (2021). Healthy diet, depression and quality of life a narrative review
of biological mechanisms and primary prevention
opportunities. World J Psychiatry.

[53] Marx W, Lane M, Hockey M, Aslam H, Berk M, Walder K (2021). Diet and depression exploring the biological mechanisms of
action. Mol Psychiatry.

[54] Sánchez-Villegas A, Álvarez-Pérez J, Toledo E, Salas-Salvadó J, Ortega-Azorín C, Zomeño MD (2018). Seafood consumption, omega-3 fatty acids intake, and life-time
prevalence of depression in the PREDIMED-plus trial. Nutrients.

[55] Berger M, Taylor S, Harriss L, Campbell S, Thompson F, Jones S (2020). Cross-sectional association of seafood consumption,
polyunsaturated fatty acids and depressive symptoms in two Torres Strait
communities. Nutr Neurosci.

[56] Molendijk M, Molero P, Ortuño Sánchez-Pedreño F, Van der Does W.Angel Martínez-González M (2018). Diet quality and depression risk a systematic review and
dose-response meta-analysis of prospective studies. J Affect Disord.

[57] Sadeghi O, Keshteli AH, Afshar H, Esmaillzadeh A, Adibi P (2021). Adherence to Mediterranean dietary pattern is inversely
associated with depression, anxiety and psychological
distress. Nutr Neurosci.

[58] Morales FJ, Mesías M, Delgado-Andrade C (2020). Association between heat-induced chemical markers and
ultra-processed foods a case study on breakfast cereals. Nutrients.

[59] Martínez-González MA, Hershey MS, Zazpe I, Trichopoulou A (2017). Transferability of the Mediterranean diet to non-Mediterranean
countries What is and what is not the Mediterranean diet. Nutrients.

[60] Hu FB (2002). Dietary pattern analysis a new direction in nutritional
epidemiology. Curr Opin Lipidol.

[61] Jacka FN, Cherbuin N, Anstey KJ, Butterworth P (2015). Does reverse causality explain the relationship between diet and
depression. J Affect Disord.

[62] Lassale C, Batty GD, Baghdadli A, Jacka F, Sánchez-Villegas A, Kivimäki M (2019). Healthy dietary indices and risk of depressive outcomes a
systematic review and meta-analysis of observational studies. Mol Psychiatry.

[63] Swainson J, Reeson M, Malik U, Stefanuk I, Cummins M, Sivapalan S (2023). Diet and depression a systematic review of whole dietary
interventions as treatment in patients with depression. J Affect Disord.

[64] Adjibade M, Assmann KE, Andreeva VA, Lemogne C, Hercberg S, Galan P (2018). Prospective association between adherence to the Mediterranean
diet and risk of depressive symptoms in the French SU VI.MAX
cohort. Eur J Nutr.

[65] Dinu M, Asensi MT, Pagliai G, Lotti S, Martini D, Colombini B (2022). Consumption of ultra-processed foods is inversely associated with
adherence to the mediterranean diet a cross-sectional study. Nutrients.

[66] Lane MM, Gamage E, Travica N, Dissanayaka T, Ashtree DN, Gauci S (2022). Ultra-processed food consumption and mental health a systematic
review and meta-analysis of observational studies. Nutrients.

[67] Trigueros R, Padilla AM, Aguilar-Parra JM, Rocamora P, Morales-Gázquez MJ, López-Liria R (2020). The influence of emotional intelligence on resilience, test
anxiety, academic stress and the Mediterranean diet A study with university
students. Int J Environ Res Public Health.

[68] Antonopoulou M, Mantzorou M, Serdari A, Bonotis K, Vasios G, Pavlidou E (2020). Evaluating Mediterranean diet adherence in university student
populations Does this dietary pattern affect students' academic performance
and mental health?. Int J Health Plann Manage.

[69] Chacón-Cuberos R, Zurita-Ortega F, Olmedo-Moreno EM, Castro-Sánchez M (2019). Relationship between academic stress, physical activity and diet
in university students of education. Behav Sci (Basel).

[70] Davies A, Wellard-Cole L, Rangan A, Allman-Farinelli M (2020). Validity of self-reported weight and height for BMI
classification a cross-sectional study among young adults. Nutrition.

[71] Moreira NF, Luz VG, Moreira CC, Pereira RA, Sichieri R, Ferreira MG (2018). Self-reported weight and height are valid measures to determine
weight status results from the Brazilian National Health Survey (PNS
2013). Cad Saúde Pública.

