# Supplementary material for: Adherence to the Mediterranean diet and depression, anxiety, and stress symptoms in Chilean university students: a cross-sectional study
Source: Cad Saude Publica. 2023 Nov 13;39(10):e00206722. doi: 10.1590/0102-311XEN206722 (PMC10645059; doi:10.1590/0102-311XEN206722)
Supplement: Supplementary file 1 [file 1678-4464-csp-39-10-EN206722-s.pdf]

**Table S1** Characteristics of the total original sample and the sample included in the present analyses.

| Variables | Total original sample | | Sample included in the present analyses | |
|---|---|---|---|---|
| | **N** | **n (%)** | **N** | **n (%)** |
| Participants | 1,234 | | 934 | |
| Women | | 658 (53.3) | | 494 (52.9) |
| Age [median (p25-p75)] | 1,234 | 19.0 (18-19) | 934 | 19.0 (18-19) |
| Maternal schooling level | 1,009 | | 934 | |
| Secondary education or less | | 671 (66.5) | | 626 (67.0) |
| Higher education | | 338 (33.5) | | 308 (33.0) |
| BMI (kg/m$^2$) | 1,180 | | 934 | |
| Underweight | | 46 (3.9) | | 40 (4.3) |
| Normal | | 691 (58.6) | | 550 (58.9) |
| Overweight | | 321 (27.2) | | 251(26.8) |
| Obesity | | 122 (10.3) | | 93 (10.0) |
| Sedentary lifestyle | 1,144 | | 934 | |
| Yes (≥ 8 hours/day sitting) | | 322 (29.0) | | 261 (27.9) |
| No (< 8 hours/day sitting) | | 812 (71.0) | | 673 (72.1) |
| Physical activity * | 1,154 | | 934 | |
| Yes | | 810 (70.2) | | 655 (70.1) |
| No | | 344 (29.8) | | 279 (29.9) |
| Tobacco use | 1,212 | | 934 | |
| Never | | 935 (77.2) | | 740 (79.2) |
| Occasional | | 215 (17.7) | | 151 (16.2) |
| Weekly or daily | | 62 (5.1) | | 43 (4.6) |
| Alcohol use | 1,212 | | 934 | |
| Never | | 396 (32.7) | | 311 (33.3) |
| Occasional | | 730 (60.2) | | 564 (60.4) |
| Weekly or daily | | 86 (7.1) | | 59 (6.3) |
| DASS-21 score [median (p25-p75)] ** | 1,234 | | 934 | |
| Depression | | 2 (1-5) | | 2 (1-5) |
| Anxiety | | 2 (1-5) | | 2(1-5) |
| Stress | | 4(2-7) | | 4 (2-7) |
| DASS-21 symptoms *** | 1,234 | | 934 | |
| Depression (> 5 points) | | 284 (23.0) | | 223 (23.9) |
| Anxiety (> 4 points) | | 342 (27.7) | | 275 (29.4) |
| Stress (> 5 points | | 450 (36.5) | | 354 (37.9) |
| Mediterranean diet score (mean ± SD) # | | 5.3 ± 1.6 | | 5.2±1.6 |
| Adherence to Mediterranean diet # | 1,167 | | 934 | |
| High/Moderate (5-14 points) | | 713 (61.1) | | 568 (60.8) |
| Low (< 5 points) | | 454 (38.9) | | 366 (39.2) |

BMI: body mass index; DASS-21: *Depression Anxiety and Stress Scale*; SD: standard deviation.
Note: data are presented as mean ± SD, median (p25-p75), or number (%).
* Physical activity according to meet recommendation of at least 5 days of moderate activity and/or walking [31];
** DASS-21 proposed by Mella et al. [27];
*** DASS-21 symptoms according to cutoff points proposed by Román et al. [28];
# Mediterranean diet adapted to Chile, according to the score proposed by Echeverria et al. [26].

**Table S2** Sensitivity analysis.

| Level of adherence | Chilean-MDI (whole) | | Chilean-MDI (whithou wine) | |
|---|---|---|---|---|
| | **OR (95%CI)** | **p-value** | **OR (95%CI)** | **p-value** |
| Low | 1.00 (Reference) | | 1.00 (Reference) | |
| Moderate/High | 0.64 (0.47-0.88) | 0.006 | 0.64 (0.47-0.88) | 0.006 |

95%CI: 95% confidence interval; Chilean-MDI: Mediterranean dietary index validated in Chile; OR: odds ratio. Note: model adjusted by sex (female, male) and age (years, continuous), mother's education level (secondary school or less, higher education), body mass index (underweight, healthy, overweight, and obese), tobacco consumption (never, occasional, weekly/daily), alcohol consumption (never, occasional, weekly or daily), sedentary lifestyle (yes, no) and physical activity (meeting recommendation: yes, no).
